# Case Report: Intractable hiccups induced by gallbladder necrosis after laparoscopic distal D2 radical gastrectomy: two cases report

**DOI:** 10.3389/fsurg.2026.1744253

**Published:** 2026-02-17

**Authors:** Zhi Zhao, Jinquan Lin

**Affiliations:** 1 Department of Gastrointestinal and Hernia Surgery, People’s Hospital of Guilin, Guilin, Guangxi, China; 2 General Surgery Department, The People’s Hospital of Cenxi City, Wuzhou, Guangxi, China

**Keywords:** gallbladder necrosis, gastrectomy, gastric cancer, intractable hiccups, postoperative complication

## Abstract

Gallbladder necrosis after gastrectomy is very rare, and intractable hiccups caused by gallbladder necrosis is even more rare. Its clinical presentations and management strategies have not been described in available literature. This report firstly describes the clinical presentations, cause, management strategies, and treatment outcome of intractable hiccups immediately after gastrectomy. When intractable hiccups occurs after gastrectomy and abdominal computed tomography (CT) indicates gallbladder enlargement, gallbladder necrosis should be considered. After cholecystectomy or ultrasound-guided percutaneous gallbladder drainage, the patients were successfully treated.

## Introduction

Gastric cancer (GC) is one of the most common cancer and the fifth leading cause of cancer-related mortality worldwide (1). Surgery remains the mainstay of treatment for locally advanced gastric cancer. Despite the developments in gastric cancer surgery aimed at improving outcomes, the radical gastrectomy is still a high-risk procedure (2–5). Studies report that the incidence of postoperative complications after D2 gastrectomy ranges from 12.8% to 14% (6–8). Major complications include hemorrhage, anastomotic leakage, lymphatic leak, pancreatic fistula, ileus and cholecystitis. Reports of hiccups after gastrectomy are very rare, particularly in intractable hiccups. Here, we first report two cases of intractable hiccups induced by gallbladder necrosis after laparoscopic distal D2 gastrectomy.

## Case presentation

### Case 1

**Figure 1:**
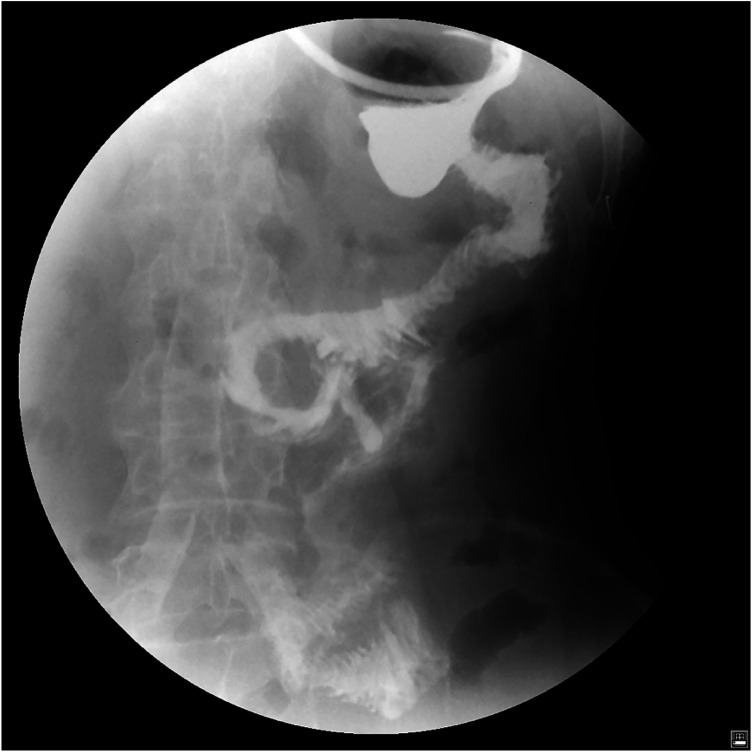
Upper gastrointestinal angiography showed no signs of anastomotic leakage.

**Figure 2:**
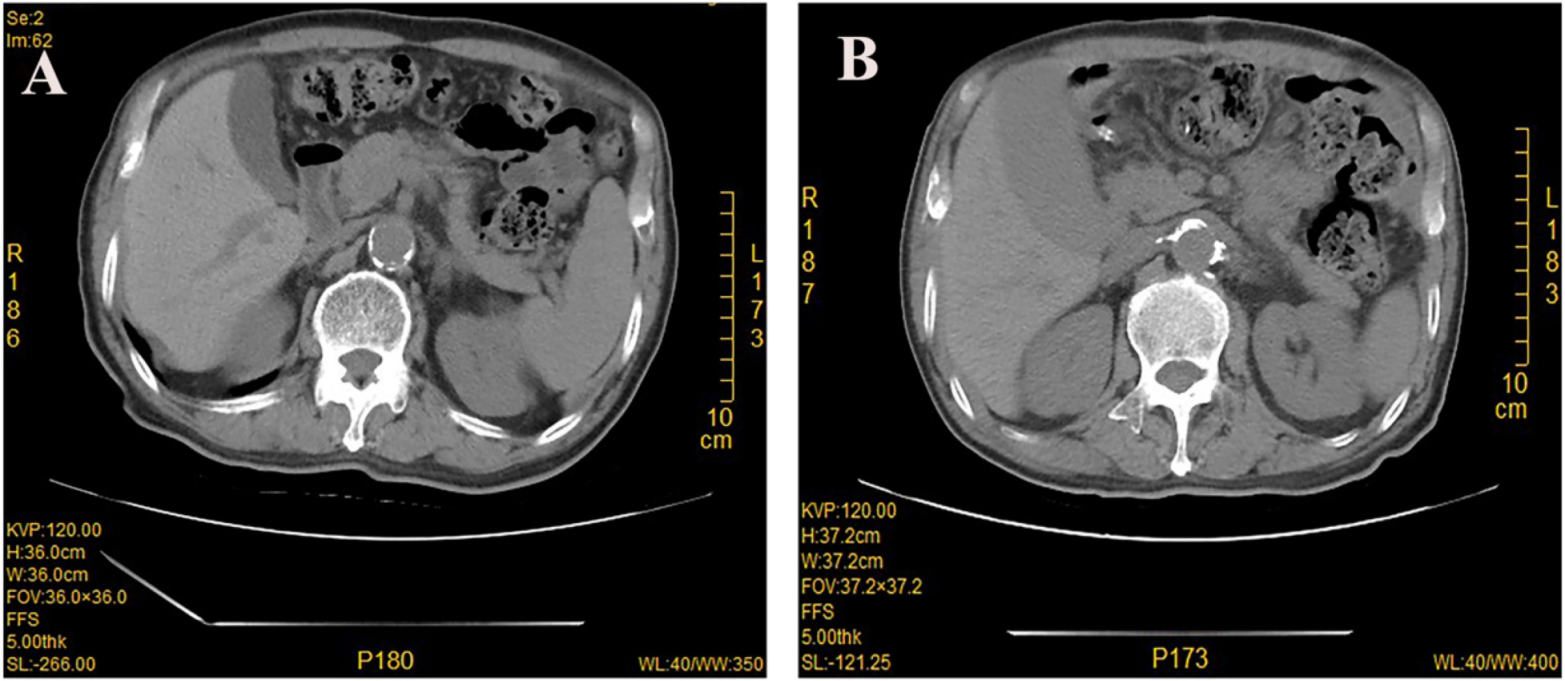
Computed tomography (CT) scan showing normal gallbladder before operation **(A)**, enlarged gallbladder on day 7 after operation **(B)**.

**Figure 3:**
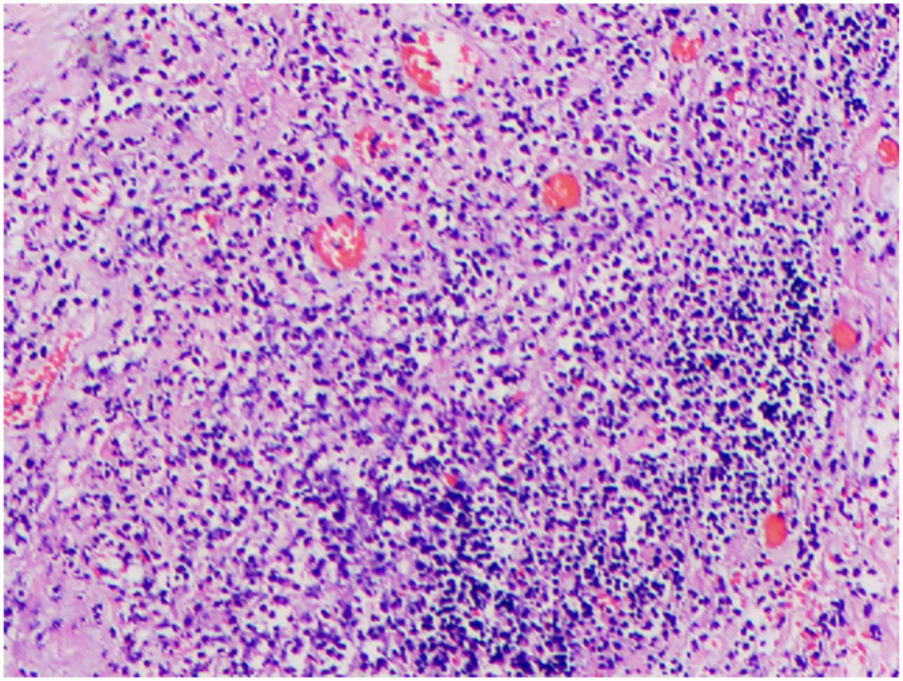
HE staining showed gallbladder necrosis.

A 77-year-old man with gastric antral adenocarcinoma underwent laparoscopic-assisted radical distal gastrectomy with D2 lymphadenectomy (Billroth II) in our department in May 2017. The patient had no preoperative history of gallstones and cholecystitis. On postoperative day 5, the patient developed intractable hiccups following eating semiliquid diet, which persisted despite conservative management including Valsalva maneuver, carotid sinus massage, and digital ocular pressure. Moreover, the patient was treated with different drugs, including metoclopramide, chlorpromazine, and herbal medicine, the symptoms also could not be relieved. Physical examination revealed right upper quadrant abdominal tenderness and muscle tension, but without rebound tenderness and Murphy's sign. Laboratory findings showed progressively elevated white blood cell count, but the bilirubin level was normally. Abdominal drainage exhibited normal ascites without early anastomotic leakage. In addition, upper gastrointestinal angiography showed no evidence of anastomotic leakage (Figure 1). Abdominal CT scan demonstrated enlarged gallbladder on day 7 after operation (Figure 2). Ten days after operation, the patient developed acute diffuse peritonitis and underwent laparotomy. Intraoperative findings revealed gallbladder necrosis and cholecystectomy was performed. The postoperative pathological results also confirmed gallbladder necrosis (Figure 3). The patient recovered well after operation and was discharged without complications.

### Case 2

**Figure 4:**
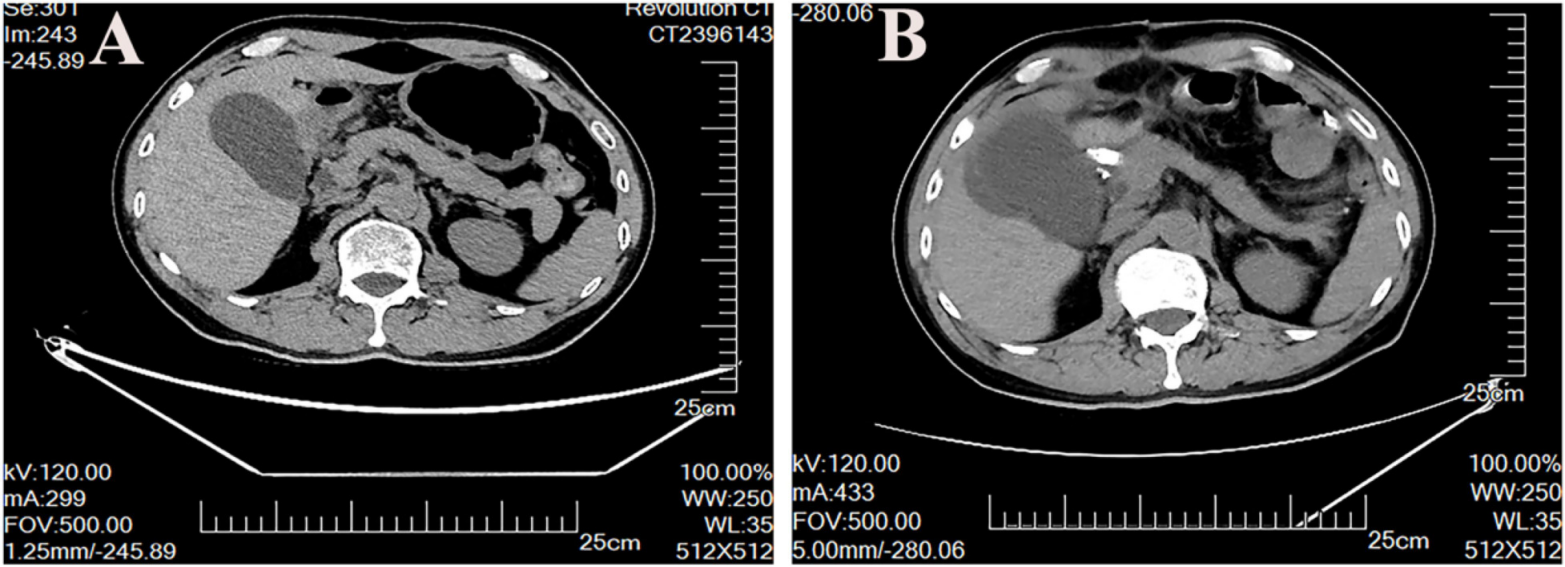
Computed tomography (CT) scan showing normal gallbladder before operation **(A)**, enlarged gallbladder on day 8 after operation **(B)**.

A 58 year-old-man with gastric antral adenocarcinoma underwent laparoscopic-assisted distal D2 gastrectomy (Billroth II) in our department in October 2023. The patient had no history of gallstones and cholecystitis. On postoperative day 4, the patient developed intractable hiccups that failed to respond to pharmacologic interventions, including metoclopramide, chlorpromazine and traditional Chinese medicine. Physical examination revealed no signs of peritonitis, and abdominal drainage tubes showed normal ascites without early anastomotic leakage. Laboratory findings demonstrated leukocytosis (WBC up to 19 × 10⁹/L) and progressive hyperbilirubinemia (total bilirubin peaked at 99 μmol/L). Abdominal CT demonstrated only gallbladder edema without subdiaphragmatic abscess or effusion (Figure 4). In view of the experience of the previous case, consultation with hepatobiliary surgeons suggested gallbladder necrosis. On postoperative day 8, ultrasound-guided percutaneous gallbladder drainage was performed. The gallbladder puncture drained purulent turbid bile. After drainage, the patient's hiccup symptoms disappeared, white blood cells and bilirubin gradually returned to normal, and was discharged without complications.

## Discussion

At present, radical gastrectomy remains the cornerstone of gastric cancer management (9–11). We have reported for the first time two cases of refractory hiccup caused by gallbladder necrosis after laparoscopic distal D2 radical gastrectomy, which provides a treatment method for other doctors encountering similar cases. In traditional beliefs, postoperative hiccups are often attributed to diaphragmatic stimulation, gastric dilation, or central nervous system factors. But the intractable hiccups caused by gallbladder necrosis provides a new perspective for general surgeons.

In these two cases of gallbladder necrosis, the first case of intractable hiccups was managed conservatively, but it showed no improvement. Laparotomy was delayed until the patient developed acute generalized peritonitis, with intraoperative identification of gallbladder necrosis. After cholecystectomy, the patient recovered well and was discharged without complications. The second patient had intractable hiccups accompanied by progressive increase in bilirubin. Abdominal CT showed gallbladder enlargement and edema. Drawing on insights from the prior case, we suspected gallbladder necrosis. After ultrasound-guided gallbladder puncture and drainage, the patient's intractable hiccups gradually resolved, bilirubin levels returned to normal, and he was discharged without complications.

It is well known that laparoscopic D2 gastrectomy necessitates the dissection of No. 8 and No. 12 lymph nodes, which may damage the cystic artery. Moreover, the anatomical variation rate of the cystic artery ranges from 10% to 35% (12). These may contribute to postoperative gallbladder necrosis. According to the literature, the incidence of cholecystitis following D2 gastrectomy ranges from 15% to 25%, even when the cystic artery is preserved intraoperatively (13, 14). This phenomenon may result from intraoperative injury to the vagus nerve during the dissection of No. 8 and No. 12 lymph nodes.

Therefore, the possible causes of intractable hiccups secondary to gallbladder necrosis are as follows: firstly, inflammation and edema of the gallbladder can stimulate the nerve endings within the gallbladder wall. These neural signals are either transmitted to the central nervous system or directly spread to the adjacent phrenic nerve branches, resulting in involuntary paroxysmal spasms of the diaphragm and subsequent onset of hiccups. Secondly, gallbladder enlargement and edema of the surrounding tissues can exert upward compression on the diaphragm, altering its normal anatomical position and tension, which in turn induces hiccups.

To prevent postoperative gallbladder necrosis, the surgeon should perform a preoperative review of abdominal CT to identify the origin of the right gastric artery. Careful intraoperative dissection is essential to avoid cystic artery injury. Postoperative gallbladder necrosis following gastric cancer surgery often presents with a delayed onset, making it difficult for surgeons to detect intraoperatively. Therefore, if the patient develops intractable hiccups, progressive elevation of bilirubin, or gallbladder edema postoperative, the possibility of gallbladder necrosis should be considered. Based on our clinical experience, ultrasound-guided gallbladder puncture and drainage is an efficient and safe treatment option, which can avoid secondary operation and is more readily to accept by the patient.

## Conclusion

Gallbladder necrosis after laparoscopic radical gastrectomy is a rare but life-threatening complication. Therefore, if a patient develops intractable hiccups postoperatively, accompanied by progressive bilirubin elevation and gallbladder edema, the possibility of gallbladder necrosis should be considered. Ultrasound-guided gallbladder puncture and drainage is an efficient and safe treatment option. To prevent such complications, carefully preoperative abdominal CT review and intraoperative dissection are essential.

## Data Availability

The original contributions presented in the study are included in the article/Supplementary Material, further inquiries can be directed to the corresponding author.
